# Treatment of Scrofulous Affections by the Preparations of Walnut Leaves

**Published:** 1842-03

**Authors:** 


*Treatment of Scrofulous affections by the preparations of Walnut leaves.*—M. Negrier of Angiers has used the above remedy in scrofulous children, nine of whom had osseous enlargements with caries; seven ulcerated glands; one several swollen cervical glands with scrofulous ophthalmia of both eyes. Each patient took daily two or three cups of infusion of bruised walnut leaves sweetened with sugar or honey, and a four-grain pill of the extract of the leaves, or a spoonful of a syrup prepared with eight grains of the same extract to ten drachms of syrup. All the sores were washed with a strong decoction of the leaves, and covered with linen compresses steeped in this decoction, or poultices made with flour and the decoction. Seven of the patients were completely cured after six months of this treatment, and five nearly so; and Mr. Negrier concludes that the walnut leaves are superior to all other antiscrofulous remedies.—*Brit. and For. Med. Rev.* Oct. 1841, from *Bull. Gén. de Thérap.* May 1841.

